# Comprehensive Analysis on Physicochemical Properties and Characteristic Compounds of Insect-Infested Ziziphi Spinosae Semen

**DOI:** 10.3390/metabo15030188

**Published:** 2025-03-11

**Authors:** Bo Xu, Zhenying Liu, Yanzhen Shen, Yunxia Cheng, Pingping Song, Feifei Wang, Zhimao Chao

**Affiliations:** 1 Institute of Chinese Materia Medica, China Academy of Chinese Medical Sciences, Beijing 100700, Chinasongpingping122@163.com (P.S.); 2 Department of Pharmacy, Beijing Health Vocational College, Beijing 101101, China; 3 Graduate School of China Academy of Chinese Medical Science, Beijing 101101, China; 4 Yunnan Botanee Bio-Technology Group Co., Ltd., Kunming 650106, Chinawangfeifei@botanee.com (F.W.)

**Keywords:** Ziziphi spinosae semen, *Plodia interpunctella*, insect infestation, qualitycharacters, volatile organic compounds, untargeted metabolomics

## Abstract

**Objectives:** Ziziphi spinosae semen (ZSS), an edible and medicinal substance, was easily infested by *Plodia interpunctella* (*P. interpunctella*) during storage. However, there was no identification method for insect-infested ZSS based on its chemical composition. Therefore, the characteristic compounds in ZSS before and after being infested by *P. interpunctella* were discovered based on the comparison of volatile organic compounds (VOCs), untargeted metabolomics, and other quality characters. **Methods:** Color, total flavonoid content (TFC), and main active compound content were measured to explore the change of physicochemical properties in ZSS after being infested by *P. interpunctella*. Non-targeted metabolomic techniques, including ultra-performance liquid chromatography–mass spectrometry (UPLC-MS) and headspace solid-phase microextraction–gas chromatography–mass spectrometry (HS-SPME-GC-MS) were used to assess molecular-level alterations. **Results:** The color changed significantly. The TFC and main active compounds of spinosin, jujuboside A, jujuboside B, and betulinic acid were decreased significantly. A total of nine VOCs and twenty-one metabolites were screened out that could be used to identify whether ZSS was infested. And some metabolites, such as uric acid, gluconic acid, hypoxanthine, and xanthine, were discovered as characteristic compounds in ZSS after being infested by *P. interpunctella*. **Conclusions:** The study provided the basis and reference for the identification of insect-infested ZSS and offered an example for the identification of other insect-infested edible and medicinal materials.

## 1. Introduction

Ziziphi spinosae semen (ZSS), the dried ripe seed of *Ziziphus jujuba* Mill. var. *spinosa* (Bunge) Hu ex H. F. Chou (Fam. Rhamnaceae), called Suanzaoren in Chinese, is one of the most common medicinal materials in clinical practice for lack of energy, restlessness, sleeplessness, heart palpitations due to fear, disturbed dreams, and frail health [1]. The primary chemical constituents of ZSS are saponins (jujuboside A et al.), flavonoids (spinosyn and swertisin et al.), alkaloids (sanjoinine B and sanjoinine C et al.), and fatty acids (linoleic acid and oleic acid et al.) [2,3]. Contemporary pharmacological research indicates that ZSS exhibits a range of therapeutic effects, such as hypnotic effects [4,5], protecting the nervous system [6,7], regulating immune function [8], and anti-tumor activity [9,10,11].

In recent years, ZSS has been developed as a health food and has gotten more and more attention in the food sector [2]. Subsequently, the market price is rising sharply. ZSS is highly susceptible to *Plodia interpunctella* (Hübner) (Lepidoptera: Pyralidae) infestation during storage, which leads to economic losses [12]. *P. interpunctella* is one of the most harmful stored-product insects, which infests medicinal materials, books, clothing, and food [13]. Newly hatched larvae move quickly and produce a lot of excrement. They carry and spread pathogenic bacteria and fungi during infestation, which have adverse effects on people’s health [14,15].

ZSS may turn into powder after being infested by *P. interpunctella*, and lose edible and medicinal value. But the insect-infested ZSS is usually crushed into powder so that consumers cannot authenticate it, and the powder is used for food material and Chinese patent drugs by a few illegal enterprises. However, there is no distinguishing method for insect-infested ZSS. Therefore, it is essential to investigate the alterations in ZSS following insect infestation and identify potential markers.

There is currently not much research on insect infestations of products. The existing literature reports mainly focus on hawthorn and jujube fruit. ^1^H-NMR and untargeted metabolomics were adopted to explore the metabolic changes in the hawthorn after insect infestation, and this adaptation has identified potential biomarkers. It was reported that color, total organic acids, total phenolics, and total flavonoids were effective indicators for quality evaluation of insect infestation, and uric acid and hippuric acid can serve as biomarkers for the deterioration of the quality of hawthorn berries during storage. For jujube fruit, gas chromatography–mass spectrometry and gas chromatography ion mobility spectroscopy technology were used to study potential biomarkers, which were the main component for determining whether red dates had been damaged by insects [16,17,18].

The study assumed that the metabolites of ZSS underwent changes after insect infestation. The objective of this research was to clarify the effects of insect infestation on the physicochemical characteristics of ZSS and explore potential biomarkers. By employing non-targeted metabolomic techniques, including ultra-performance liquid chromatography–mass spectrometry (UPLC-MS) and headspace solid-phase microextraction–gas chromatography–mass spectrometry (HS-SPME-GC-MS), the study aimed to assess molecular-level alterations. To investigate whether there was a significant change in the content of metabolites, *t*-test was used for examination. Principal component analysis (PCA) is an unsupervised statistical method for reducing the dimensions of a database by using linear combinations of a starting set of variables based on their maximum variance, which can convert original variables into new independent variables named principal components (PCs). It can make a preliminary judgment on the distribution status, natural aggregation, and abnormal samples. Orthogonal partial least squares-discriminant analysis (OPLS-DA) extends the regression of PCA, uses class membership to maximize the variation, and introduces an orthogonal signal correction filter to separately handle the systematic variation that is correlated or uncorrelated to the Y variable. It has a better discriminant ability for the samples with larger within-class divergence than that of PCA. Therefore, *t*-test, PCA, and OPLS-DA were adopted for data analysis.

## 2. Materials and Methods

### 2.1. Materials and Treatments

Normal ZSS was collected from Hebei Province on 25 December 2020 and identified as the dried ripe seeds of *Ziziphus jujuba* Mill. var. *spinosa* (Bunge) Hu ex H. F. Chou (Fam. Rhamnaceae) by Prof. Zhimao Chao (Institute of Chinese Materia Medica, China Academy of Chinese Medical Sciences). The reference specimens (No. ZSS20201225) were stored at 1022 Laboratory of the Institute of Chinese Materia Medica, China Academy of Chinese Medical Sciences, Beijing.

**Figure 1:**
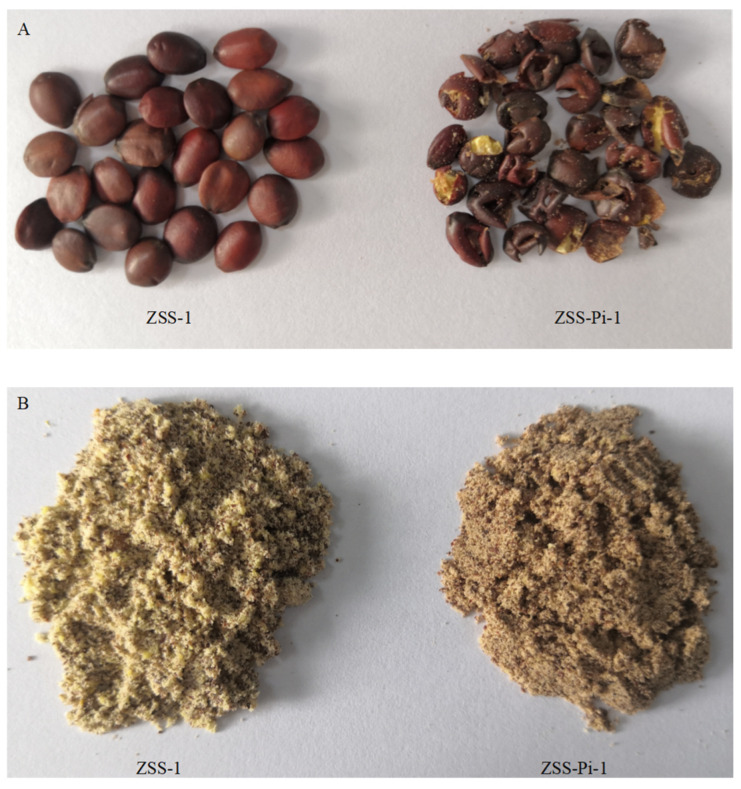
Samples of ZSS-1 and ZSS-Pi-1 (**A**) and powder of ZSS-1 and ZSS-Pi-1 (**B**).

All ZSS were randomly divided into six groups. Three groups (25 g) acted as normal ZSS for the control group (ZSS group, ZSS-1~ZSS-3). The other three groups acted as insect-infested ZSS for the experimental group (ZSS-Pi group, ZSS-Pi-1~ZSS-Pi-3), in which 75 larvae of *P. interpunctella* and 25 g ZSS were put into a 250 mL beaker together and placed in a ZSW-HQ150 comprehensive strong light stability test box (Zhenxiang Electromechanical Technology Shanghai Co., Ltd.; Shanghai, China) at 25 °C with a relative humidity of 55%. After being cultured for 20 days, ZSS was seriously infested by *P. interpunctella* in the experimental group, and all the insects were taken out. Both normal and insect-infested samples were crushed into powder (DFT-50, Linda Machinery Co. Ltd.; Hangzhou, China), and passed through a 24-mesh sieve. The samples of ZSS-1 and ZSS-Pi-1 are shown in Figure 1.

### 2.2. Quality Characters

#### 2.2.1. Color Measurement

The color parameters of L*, a*, and b* were assessed with an NH310 colorimeter (Shenzhen three NH Technology Co., Ltd.; Guangdong, China). The total color difference (Δ*E*) was calculated from ΔL*, Δa*, and Δb* as follows: ΔE* *=* (ΔL*^2^ + Δa*^2^ + Δb*^2^)^1/2^. The color difference meter was calibrated using the instrument’s white tile standard. A powder sample of 2.8 g was put into the test box and measured with the colorimeter. The samples were analyzed three times repetitively, and the results were expressed as a mean ± standard deviation (SD) [19,20].

#### 2.2.2. Total Flavonoid Contentf

The total flavonoid content (TFC) was determined using the NaNO_2_-Al(NO_3_)_3_-NaOH method [21]. A powder sample of 1.0 g was mixed with 40 mL of 70% aqueous ethanol (Fuyu Fine Chemical Co., Ltd.; Tianjin, China), and extracted with a KQ-100E ultrasonicator (100 W and 40 kHz, Kunshan Ultrasonic Instruments Co. Ltd.; Kunshan, China) for 30 min. An aliquot of a 6 mL, 15-fold diluted extract solution was put into a 25 mL flask. The mixture was added sequentially with a 1 mL 5% NaNO_2_ solution (Fuchen (Tianjin, China) Chemical Reagent Co., Ltd.; Tianjin, China), a 1 mL 10% aluminum nitrate solution , and 10.0 mL 4% sodium hydroxide solution at 0, 6, and 12 min. Ultrapure water was immediately added into the flask to a volume of 25 mL and stood for 15 min. The absorbance was measured at 500 nm with a TU-1810ASPC UV-visible spectrophotometer (Purkinje General Instrument Co. Ltd.; Beijing, China). Samples were analyzed three times repetitively. A series of rutin standard solutions (8.006~48.04 μg/mL) were used for the calibration curve. The linear regression equation for the absorbance under the concentrations was as follows: *Y* = 11.361 *X* −0.0081, *R*^2^ = 0.998. The TFC was calculated as mg rutin equivalent per g sample (mg/g).

#### 2.2.3. Main Active Compound Content

##### Chromatographic Condition

The content of spinosin, jujuboside A, jujuboside B, and betulinic acid was determined with an analytical Shimadzu LC-20AT apparatus (Shimadzu Corporation; Tokyo, Japan), which was equipped with an Agilent ZORBAX SB C_18_ column (4.6 mm × 250 mm, 5 µm). The mobile phase consisted of 0.1% formic acid/water (*v*/*v*, A) and acetonitrile (B). The gradient elution was performed as the following protocol: 0~6 min, 0~14% B; 6~13 min, 14~33% B; and 13~22 min, 33~80% B. The flow rate was 1.0 mL/min. The injection volume was 10 µL and the column oven temperature was set at 35 °C. The detection wavelength was selected at 335 nm for spinosin, 210 nm for betulinic acid, and 203 nm for jujuboside A and jujuboside B.

##### Preparation of Sample Solution

A 1.0 g powder sample was precisely weighed, combined with 25 mL of a 70% ethanol solution, and weighed again. It was then subjected to an ultrasonic extraction for 30 min (100 W, 40 kHz). After cooling, the sample was re-weighed, and any weight loss was compensated with a 70% ethanol solution. The mixture was filtered through filter paper. A 15 mL portion of the filtrate was concentrated under reduced pressure at 50 °C, and the resulting dried extract was dissolved in 5 mL of methanol to form the sample solution. Finally, the solution was filtered through a 0.22 µm membrane filter prior for HPLC analysis.

##### Preparation of Standard Solution

A mixed standard solution of spinosin, jujuboside A, jujuboside B, and betulinic acid was prepared with methanol to a final concentration of 10.96, 25.20, 25.08, and 13.23 mg/mL, respectively. The standard solution was filtered through a 0.22 µm membrane filter before HPLC analysis. Samples were analyzed three times repetitively.

##### Method Validation

The precision was estimated by six consecutive injections of a standard solution. The stability was evaluated using a sample solution stored at 25 °C for different time intervals: 0, 2, 4, 8, 12, 24, and 48 h. Meanwhile, the repeatability was assessed by conducting six replicate analyses of the same sample. A known amount of mixed standard solution was added to the quantified sample (0.50 g), and the recovery rate was analyzed using the spiked sample. The linearity was established via a calibration curve of peak area versus concentration [22].

### 2.3. Volatile Organic Compounds

#### 2.3.1. Sample Preparation

The volatile organic compounds (VOCs) in ZSS and ZSS-Pi samples were extracted using the headspace solid-phase microextraction (HS-SPME) technique. Specifically, 0.5 g of the powder sample was precisely weighed and placed into a 20 mL headspace vial with a polytetrafluoroethylene (PTFE) septum. The sample was then preheated at 90 °C for 10 min. An SPME fiber (50/30 μm PDMS/DVB/CAR) was inserted through the needle and exposed to the headspace of the vial to adsorb VOCs for 40 min. Afterward, the fiber was quickly transferred and injected into the gas chromatography injection port at 250 °C for 5 min to desorb the VOCs [23].

#### 2.3.2. Analysis Condition

The analysis of VOCs was conducted using gas chromatography-mass spectrometry (GC-MS). The instrumentation included a Shimadzu GC-MS-QP 2010 Plus gas chromatograph-mass spectrometer coupled with a Swiss CTC Combi-xt PAL three-in-one multifunctional autosampler. The separation was achieved on a UI elastic quartz capillary column (0.25 µm × 0.25 mm × 30 m, Agilent, Palo Alto, CA, USA). The splitless injection mode was employed, and high-purity helium was used as the carrier gas at a constant flow rate of 1 mL/min. The temperature program was set as follows: the initial temperature was maintained at 50 °C for 5 min, then ramped to 100 °C at a rate of 10 °C/min and held for 5 min, followed by an increase to 300 °C at 25 °C/min and held for 4 min. The ion source temperature was 200 °C, the interface temperature was 230 °C, the solvent delay time was 2 min, and the scanning range was *m*/*z* 45~500. The mass spectra were obtained at 70 eV with electron ionization (EI) mode [24]. The samples were analyzed three times repetitively.

#### 2.3.3. Data Processing

The primary identification of mass spectra was conducted by comparing the data with the National Institute of Standards and Technology (Gaithersburg, MD, USA) (NIST) 14 database. The qualitative analysis was confirmed by matching the retention time and mass spectra. The quantitative analysis was performed using the sum of peak area normalization method based on the total ion chromatogram, with the results processed using GCMS solution software (version 4.30) [25,26].

#### 2.3.4. Statistical Analysis

The results were presented as mean ± SD, and statistical significance was determined using Student’s *t*-test (*p* < 0.05) via SPSS software (version19.0, IBM, Armonk, NY, USA). Multivariate analysis and calculations were performed using SIMCA-p software (Version 14.1, Umetrics, Malmö, Sweden). PCA was used to identify outliers, and ensure the clustering of quality control (QC) samples, which revealed inherent sample groupings. Meanwhile, OPLS-DA was employed to pinpoint the variables contributing to class separation. The differential compounds between ZSS and ZSS-Pi groups were filtered with variable importance in the projection (VIP) and S-plot values. A heat map was generated with the MetaboAnalyst 5.0 website (https://www.metaboanalyst.ca/) (accessed on 24 December 2021) to intuitively show the content gap of differential compounds [27,28].

### 2.4. Untargeted Metabolomics

#### 2.4.1. Preparation of Sample Solution

A powder sample of 100 mg was weighed accurately and extracted with 0.5 mL 80% aqueous methanol by a UVS-1 well vortex (Beijing Yousheng United Technology Co., Ltd.; Beijing, China). The mixture was kept on ice for 5 min and then centrifuged at 15,000× *g* for 20 min at 4 °C. A portion of the supernatant was diluted to a final concentration of 53% methanol in water and filtered through a 0.22 μm membrane for subsequent LC-MS/MS analysis.

The QC sample solution was created by combining equal volumes of each sample extract to monitor the consistency and stability of the analytical system. A 53% methanol solution in water was used as a blank control, and its response was subtracted during data processing to correct for background interference.

#### 2.4.2. Analysis Condition

Metabolomics analysis was conducted using a UPLC system (ThermoScientific; Waltham, MA, USA) coupled with a Hypesil Gold column (1.9 μm, 100 mm × 2.1 mm, Thermo Fisher, Waltham, MA, USA) and an Orbitrap Q Exactive™ HF-X mass spectrometer (Thermo Fisher, Dreieich, Germany). In positive ion mode, the mobile phase included 0.1% formic acid in water (*v*/*v*, A) and methanol (B). In negative ion mode, the mobile phase consisted of a 5 mM ammonium acetate solution in water (A) and methanol (B). The gradient elution was set as follows: 0–1.5 min, 2% B; 1.5–12.0 min, 2–100% B; 12.0–14.0 min, 100% B; 14.0–14.1 min, 100–2% B; and 14.1–17.0 min, 2% B. The flow rate was maintained at 0.2 mL/min, and the injection volume was 2.0 µL [29].

Mass spectra were acquired with an electrospray ionization (ESI) source in both positive and negative modes with the range of mass *m/z* 100~1500. The ESI parameters were as follows: spray voltage of 3.2 kv, capillary temperature of 320 °C, sheath gas flow rate of 40 arb, and aux gas flow rate of 10 arb. Samples were analyzed three times repetitively.

#### 2.4.3. Data Processing

The raw data files were processed using Compound Discoverer 3.1 (ThermoScientific; Waltham, MA, USA) for peak alignment, extraction, and compound identification. The key parameters were configured as follows: retention time tolerance of 0.2 min, mass tolerance of 5 ppm, signal intensity tolerance of 30%, signal-to-noise ratio of 3, and minimum intensity of 100,000. Internal standard peaks, as well as any known false positive peaks (including noise, column bleed, and derivatized reagent peaks), were removed from the raw data files, deredundant and peak pooled. At least 80% of the metabolic features detected in any set of samples were retained. A threshold of 10 ppm was set in each rating criterion, which included a first-level quality deviation. Matches with fragment similarity greater than 30 points were considered valid matches. After filtering, for specific samples with metabolite levels below the lower limit of quantification, the minimum metabolite value was estimated, and each metabolic signature was normalized to the sum. To reduce the errors caused by sample preparation and instrument instability, the response intensities of the sample mass spectrometry peaks were normalized using the sum normalization method to obtain the normalized data matrix. The detected peaks were compared against the mzCloud (https://www.mzcloud.org/) (accessed on 30 December 2021), mzVault, and Masslist databases to achieve relative quantitative results. Molecular formulas were inferred from molecular ion peaks, adduct ions, and fragment ions. The peak intensities were normalized relative to the total spectral intensity.

#### 2.4.4. Statistical Analysis

Multivariate statistical analyses of PCA and OPLS-DA was performed on the SIMCA-p software. The differential metabolites between ZSS and ZSS-Pi groups were filtered with VIP. A heat map was created to visually illustrate the differences in compound levels across various samples [30,31]. The KEGG (Kyoto Encyclopedia of Genes and Genomes) database was employed for the annotation and enrichment analysis of these differential metabolites [32].

## 3. Results

### 3.1. Changes in Color

Color is important for quality evaluation and is closely related to intrinsic substances. To a certain extent, color change can reflect their quality difference [33,34]. In the study, the color parameters of ZSS and ZSS-Pi groups were measured and shown in Table 1. The results show that there was a significant difference in L^*^, a^*^, b^*^, and ΔE* between ZSS and ZSS-Pi groups (*p* < 0.05), which indicated that the color changed significantly after ZSS was infested by *P. interpunctella*. Furthermore, the results showed the lower L*, higher a*, and lower b* values in ZSS-Pi group.

### 3.2. Changes in TFC

Flavonoids are the main active ingredients in ZSS with various pharmacological activities, such as calming, anti-anxiety, anti-depression, enhancing memory, and protecting the brain and nerves [8]. The TFC was compared in the ZSS and ZSS-Pi groups and the results were shown in Table 1. Compared with ZSS group, the TFC in the ZSS-Pi group significantly decreased by 31.5% (*p <* 0.05).

### 3.3. Changes in Main Active Compound Content

The HPLC method was used to measure the content of spinosin, jujuboside A, jujuboside B, and betulinic acid simultaneously in the ZSS and ZSS-Pi groups. Calibration curves were generated using six different concentrations of standard solutions. Acceptable linear correlation was confirmed by the correlation coefficients for these four compounds, as shown in Table 2. The relative standard deviations (RSD) for stability, repeatability, precision, and recovery rate were all below 3.0%, which suggested that the sample solution, experimental method, and instrument system were reliable and stable. The method’s accuracy was assessed through recovery tests, with mean recovery rates falling within a reasonable range of 97.54% to 102.73%. The content results in the ZSS and ZSS-Pi groups were shown in Table 1. Compared with the ZSS group, the content of the four compounds decreased by 30.71%, 51.89%, 41.79%, and 14.77%, respectively (*p <* 0.05) in the ZSS-Pi group.

### 3.4. Changes in VOCs

**Figure 2:**
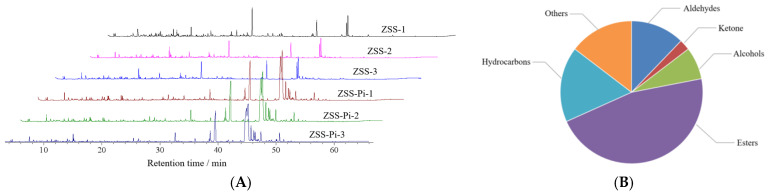
The total ion chromatograms (TIC) of the ZSS and ZSS-Pi groups (**A**). The classification of 41 volatile organic compounds (VOCs) (**B**).

The odor is an important factor that affects the life activities of insects, and volatile substances can induce their feeding and migration behaviors [12,35,36]. Therefore, HS-SPME-GC-MS can be used to analyze VOCs and their content [37,38]. The total ion chromatograms (TIC) of the ZSS and ZSS-Pi groups are shown in Figure 2A. It showed that there was no significant difference among the three ZSS samples (ZSS-1~ZSS-3), nor the three ZSS-Pi samples (ZSS-Pi-1~ZSS-Pi-3). But there was significant difference between the ZSS and ZSS-Pi groups, which indicated that the content of VOCs was changed after ZSS was infested by *P. interpunctella*.

**Figure 3:**
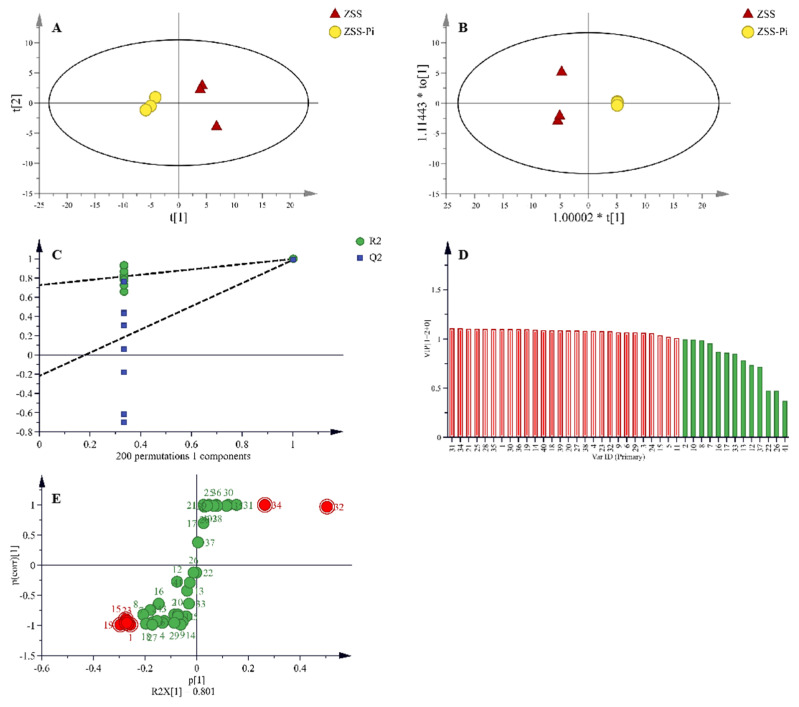
PCA (**A**) and OPLS-DA (**B**) score plots of VOCs in the ZSS and ZSS-Pi groups. Permutation tests (**C**), VIP (**D**), and S-plot of OPLS-DA model (**E**).

In total, 41 VOCs were identified based on mass spectra and retention index (RI). The calibration table and its discussion are shown in Table S1. In order to explore the difference in VOCs between the ZSS and ZSS-Pi groups, multivariate statistical analysis of PCA and OPLS-DA were carried out [39]. The PCA score plot is shown in Figure 3A. The contribution rates of PC1 and PC2 were 78.3% and 14.5%, respectively. The total contribution rate of PC1 and PC2 was 92.8%. *R*^2^ and *Q*^2^ were usually used to evaluate the quality and reliability of the PCA model. Generally, their values close to 1.0 indicated excellent fitness and predictive capability of the model [40]. In this study, the *R*^2^ and *Q*^2^ values were 0.928 and 0.806, respectively, demonstrating the stability and reliability of the PCA model. The ZSS samples were located on the right side of the *Y*-axis, while the ZSS-Pi samples were on the left side. This distribution indicated a significant difference in VOCs between the ZSS and ZSS-Pi groups.

Given that PCA might not fully eliminate within-group variations and irrelevant random errors, OPLS-DA was employed to further investigate the differences [41]. The score plot displayed a distinct separation, as shown in Figure 3B. The *R*^2^ value of 0.949 indicated a well-fitted model, while the *Q*^2^ value of 0.988 suggested strong predictive power. The permutation tests were performed 200 times, and the result of intercept *Q*^2^ was lower than 0.05 (Figure 3C), which indicated that the model was statistically valid and did not over-fit [42].

In the OPLS-DA S-plot, covariance (*p*) reflects the contribution of each variable to the variance among observations, while correlation *p* (corr) indicates the correlation between samples and the reliability of the results [43]. In this study, the criteria for screening differential compounds included |*p*| ≥ 0.8, |*p* (corr)| ≥ 0.98 in the S-plot (Figure 3D), VIP > 1 (Figure 3E), and *t*-test (*p* < 0.05). Based on these criteria, nine compounds were identified, namely methyl stearate, methyl myristate, methyl pentadecanoate, dimethyl suberate, dimethyl azelate, methyl palmitoleate, methyl oleate, 2,3-butanediol, and tetradecane.

**Figure 4:**
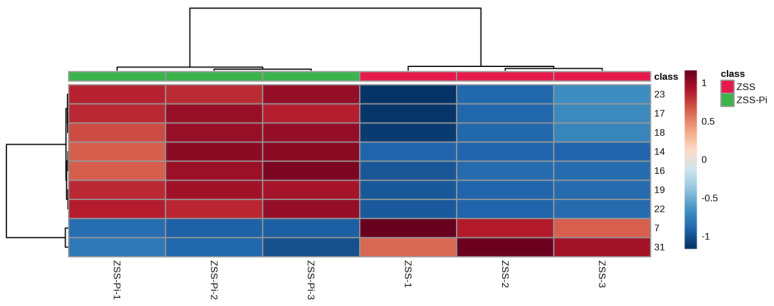
Heat map of the nine differential compounds’ content in ZSS and ZSS-Pi groups. The numbers of the heat map were consistent with the No. row in Table S1.

The content of the nine differential compounds in ZSS and ZSS-Pi groups was visualized in a heat map in Figure 4. Horizontal columns represented different samples, and vertical columns represented differential compounds. The intensity of red indicates higher content, while the intensity of blue indicates lower content. As depicted in Figure 4, the six samples were distinctly divided into two groups: the three ZSS samples were clustered on the right, and the three ZSS-Pi samples were grouped on the left. The content of 2,3-butanediol and tetradecane decreased in the ZSS-Pi group, and the content of the other seven differential compounds in all of the methyl ester, increased.

### 3.5. Metabolomic Profiling of ZSS and ZSS-Pi Samples

**Figure 5:**
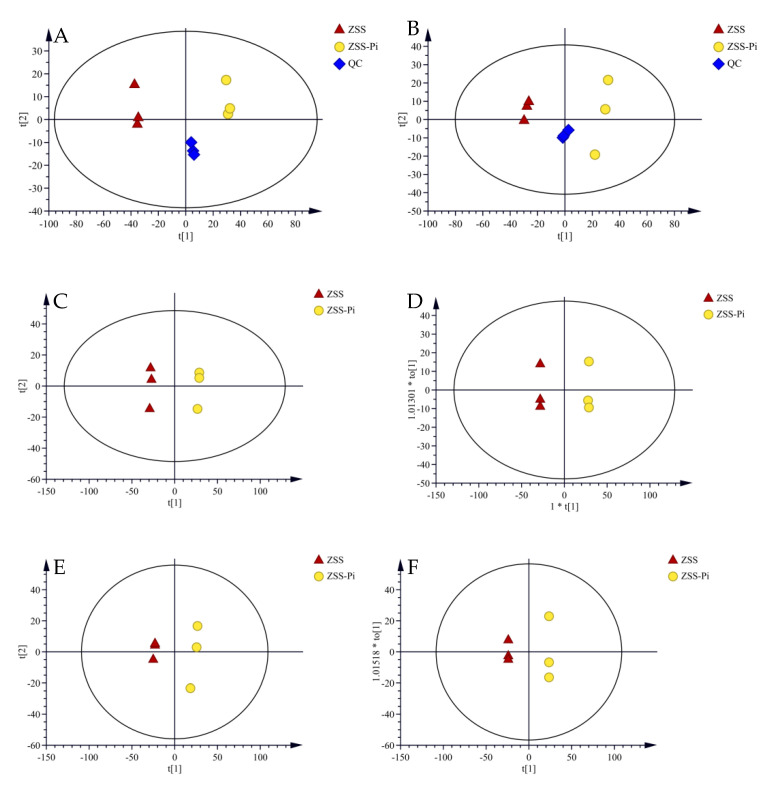
PCA score plots of ZSS, ZSS-Pi, and QC samples in the positive (**A**) and negative (**B**) modes. The PCA score plots of the ZSS and ZSS-Pi samples in positive (**C**) and negative (**D**) modes. The OPLS-DA score plots of the ZSS and ZSS-Pi samples in positive (**E**) and negative (**F**) modes.

Untargeted metabolomics combined with chemometrics have become an important and valuable method in various research [44,45]. UPLC-QE-MS was useful for the analysis of various metabolites for high selectivity and sensitivity [31]. Therefore, it was used to validate the chromatographic and mass detection system for data reliability [16]. In PCA score plots, QC samples were close to the center of the plots, confirming that the stability and reproducibility of the experiment were reliable (Figure 5A,B).

After raw data were pre-processed, two separate data matrices containing 1406 features for the positive mode and 1109 features for the negative mode were obtained. Unsupervised PCA was performed to observe the overall clustering effect and classification trend. The result is presented in Figure 5C,D. It showed that *R*^2^ and *Q*^2^ were 0.709, 0.539 for the positive mode and 0.773, 0.664 for the negative mode, indicating that the PCA models were stable and reliable. The ZSS samples were distributed on the left, and the ZSS-Pi samples were distributed on the right of the *Y* axis, suggesting that the metabolites were significantly different between the ZSS and ZSS-Pi groups.

Supervised OPLS-DA was used to further screen the differential metabolites between the ZSS and ZSS-Pi groups. It showed that *R*^2^ and *Q*^2^ were 0.773, 0.664 for the positive mode and 0.773, 0.992 for the negative mode. The score plots (Figure 5E,F) showed a clear separation of the ZSS and ZSS-Pi groups, which was consistent with the classification of the PCA models.

**Figure 6:**
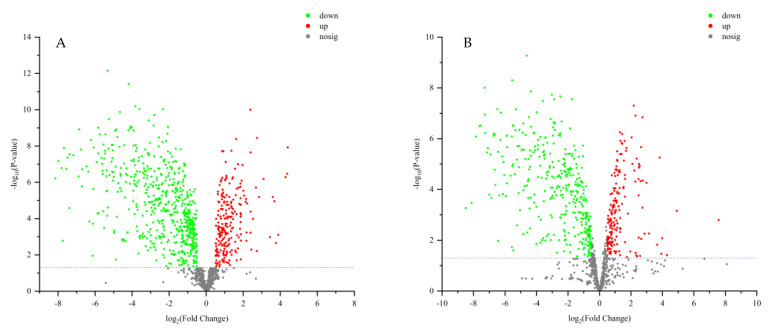
Volcano plot analysis for differential metabolite expression in positive (**A**) and negative (**B**) modes. Log_2_ fold change as the *X* axis, the multiple changes in each metabolite, and −log_10_ (*p* value) as the *Y* axis. The significantly up-regulated metabolites were shown in red, while the significantly down-regulated metabolites were shown in green, and the non-significantly different metabolites were shown in gray.

**Figure 7:**
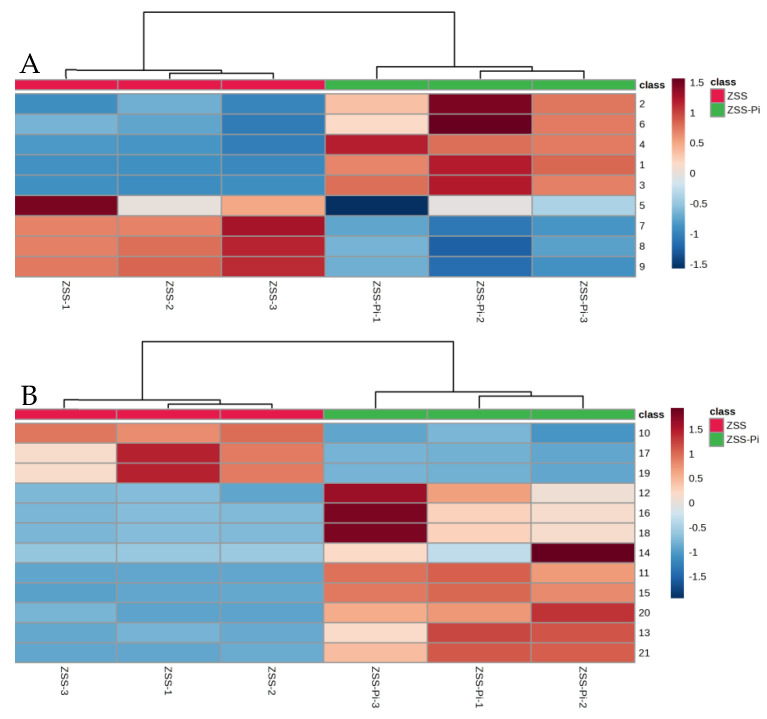
Heat maps of the differential metabolites’ content in ZSS and ZSS-Pi groups in positive (**A**) and negative (**B**) modes. The numbers in the heat map are consistent with the No. row in Table S2.

VIP > 2 in the OPLS-DA model, *p <* 0.05 in the *t*-test, and |log_2_ fold change| > 0.5 were used to screen differential metabolites. The results were shown in volcano plots (Figure 6). As a result, nine and twelve metabolites for positive and negative modes were selected (Table S2). And the content distribution of the twenty-one differential metabolites in ZSS and ZSS-Pi groups was visualized in a heat map (Figure 7A,B). As shown, six samples were clearly separated into two groups, i.e., three ZSS samples were classified into a group on the left, and three ZSS-Pi samples were classified on the right and the content of jujuboside A significantly decreased in the ZSS-Pi group, which was consistent with the result of HPLC.

**Figure 8:**
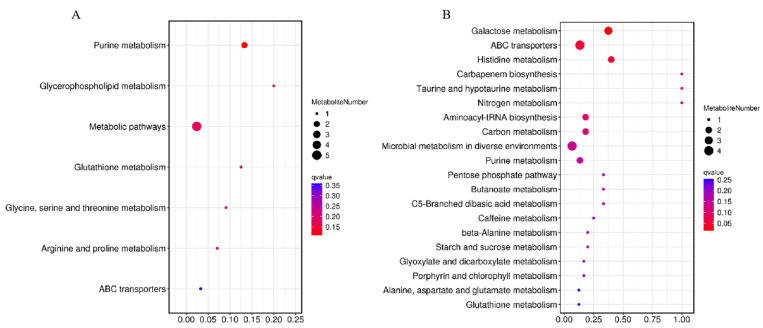
Metabolic pathway enrichment maps of differential metabolites in positive (**A**) and negative (**B**) modes. The horizontal coordinate was the enrichment significance *p*-value and the vertical coordinate was the KEGG pathway. The size of bubbles in the graph represented the number of metabolites enriched to the metabolic set in that pathway.

**Figure 9:**
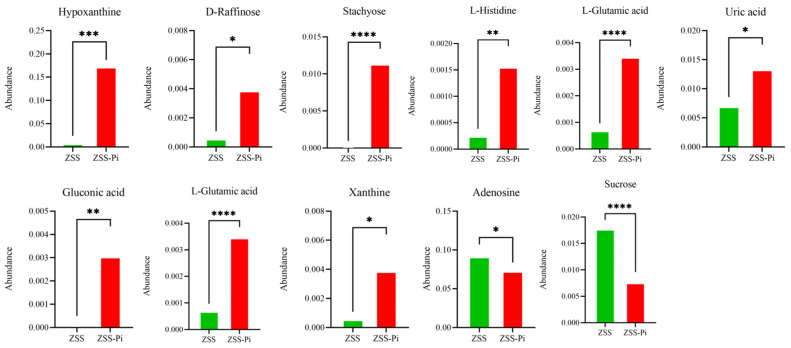
Differential metabolite abundances in ZSS and ZSS-Pi groups. (*: *p* ≤ 0.05, **: *p* ≤ 0.01, ***: *p* ≤ 0.001, ****: *p* ≤ 0.0001).

The pathways of differential metabolites were comprehensively analyzed. Seven and twenty metabolic pathways were identified in positive and negative modes, respectively (Figure 8). For the positive mode, a pathway of purine metabolism was the most significant and obvious, involving differential metabolites of hypoxanthine and adenosine. For negative mode, the pathways of galactose metabolism, ABC transporters, histidine metabolism, carbapenem biosynthesis, taurine and hypotaurine metabolism, nitrogen metabolism, aminoacyl-tRNA biosynthesis, carbon metabolism, and microbial metabolism in diverse environments were significantly enriched, involved differential metabolites of hypoxanthine, sucrose, D-raffinose, stachyose, L-histidine, L-glutamic acid, uric acid, gluconic acid, L-glutamic acid, adenosine, and xanthine. The abundance changes in the above metabolites are shown in Figure 9.

All eleven metabolites’ abundances listed above were significantly different between the ZSS and ZSS-Pi groups according to the heat map and Table S2. The abundance of hypoxanthine, D-raffinose, stachyose, L-histidine, L-glutamic acid, uric acid, gluconic acid, L-glutamic acid, and xanthine increased significantly in the ZSS-Pi group. And the other two metabolites of adenosine and sucrose decreased significantly.

## 4. Discussion

The color results showed the lower L*, higher a*, and lower b* values in the ZSS-Pi group. L* indicates lightness, a* represents redness (+) and greenness (-), and b* indicates yellowness (+) and blueness (−). Combined with the actual process of *P*. *interpunctella* infesting ZSS, the insects usually firstly break through the purple-red testa first and infest the pale-yellow cotyledons internally, resulting in an increase in purple-red testa proportions and a decrease in pale-yellow cotyledons proportion in samples. Therefore, the redness deepened, the yellowness lightened, and the lightness darkened in the ZSS-Pi samples.

TFC in the ZSS-Pi group significantly decreased by 31.5% (*p <* 0.05), indicating that insect infestation can decrease the quality of ZSS. Flavonoids are the main active ingredients in ZSS with various pharmacological activities, such as calming, anti-anxiety, anti-depression, enhancing memory, and protecting the brain and nerves [8]. The significant decrease in TFC will inevitably lead to a decrease in the clinical efficacy of ZSS. It was reported that TFC of hawthorn berries was significantly decreased after insect infestation relative to no insect-infested samples, and TFC was significantly lower in high insect density than those in low insect density, which might be due to the destruction of the cell structure of products by insects [16,17].

Previous research has indicated that insect infestation can alter the levels of active compounds and reduce the quality, nutritional value, and health benefits of the material. After infestation, the active compounds of neochlorogenic acid, chlorogenic acid, cryptochlorogenic acid, epicatechin, hyperoside, and isoquercitrin decreased relative to the no insect-infested hawthorn samples [46]. When Farfarae Flos was infested by insects, the levels of active compounds such as chlorogenic acid, isochlorogenic acid, and tussilagone were reduced, inevitably compromising its quality and clinical efficacy [47]. Likewise, insect infestation in ZSS-Pi samples led to a decrease in the content of four active compounds with the HPLC results, suggesting a decline in both quality and medicinal value. This might be due to the absorption and metabolism of saponins in ZSS by *P. interpunctella.*

ZSS was abundant with fatty acids. The results of HS-SPME-GC-MS showed that the content of 2,3-butanediol and tetradecane decreased in the ZSS-Pi group, and the content of the other seven differential compounds, including all the methyl ester, increased. It was speculated that when *P. interpunctella* infested ZSS, fatty acids were ingested and metabolized into ester compounds with related enzymes in the body of *P. interpunctella.* Therefore, the content of ester compounds increased, which provided a basis and reference for the identification of insect-infested ZSS. In view of this, it was thought that methyl esters could be chosen as the characteristic marker of ZSS infested by *P. interpunctella* and used to identify whether ZSS was infested.

It was speculated that sucrose was ingested and metabolized with related enzymes in the body of P. interpunctella for life activities [48]. The increase in organic acids’ abundance might have resulted from the metabolization of *P. interpunctella*. For example, uric acid was the final product of insect nitrogen metabolism. It had been reported that the uric acid level could be used to judge the degree of insect infestation [49,50]. These metabolites with significant differences in the ZSS and ZSS-Pi groups could be used as characteristic indexes for the identification of insect-infested ZSS, which provided references and examples for the determination of edible and medicinal materials.

However, the study only analyzed the physical and chemical characteristics, VOCs, and metabolites, and further explored the impact of insect infestation on the quality of ZSS and the changes in metabolites. A reasonable and mature identification method has not yet been established. Therefore, in subsequent research, standards for the content of biomarkers can be set for the determination of insect infestation with HPLC or UPLC, which can be used for the determination of insect infestation. Additionally, future research could delve into employing multi-omics approaches, including proteomics and transcriptomics, to elucidate the gene regulatory networks of key metabolites impacted by insect infestation. This would enhance the comprehension of the mechanisms underlying insect infestation and offer novel, environmentally friendly strategies to mitigate it.

## 5. Conclusions

In the study, a comprehensive evaluation of quality characters, VOCs, and untargeted metabolomics of ZSS before and after insect infestation was carried out. The results of quality characters showed that the color changed and the content of total flavonoid and main active compounds of spinosin, jujuboside A, jujuboside B, and betulinic acid declined significantly after ZSS was infested by *P. interpunctella*. The number of 41 VOCs were identified, of which 29 VOCs occurred significantly different in content between the ZSS and ZSS-Pi groups with HS-SPME-GC-MS. Nine differential compounds were screened out, among which seven compounds with increased content were all methyl esters. Untargeted metabolomics showed that the content of sucrose was decreased and content of several organic acids such as uric acid, gluconic acid, and azelaic acid was increased significantly. These characteristic compounds could be used for the identification of insect-infested ZSS. The related pathways include purine metabolism, galactose metabolism, ABC transporters, histidine metabolism, and so on.

It was the first time exploring the change and related pathways of ZSS before and after being infested by *P. interpunctella*, which provided a basis and reference for the identification of insect-infested ZSS and offered an example for the identification of other edible and medicinal materials.

## Figures and Tables

**Table 1:** Quality characters of ZSS and ZSS-Pi groups.

Item	ZSS	ZSS-Pi
L*	67.42 ± 1.52 *	61.84 ± 1.00
a*	4.67 ± 0.14	6.39 ± 0.18 *
b*	12.74 ± 0.70 *	11.16 ± 0.21
ΔE*	29.36 ± 0.91	33.64 ± 0.92 *
TFC (mg/g)	9.89 ± 0.97 *	6.77 ± 0.71
Spinosin (mg/g)	1.40 ± 0.25 *	0.97 ± 0.13
Jujuboside A (mg/g)	1.06 ± 0.18 *	0.51 ± 0.10
Jujuboside B (mg/g)	0.67 ± 0.17 *	0.39 ± 0.08
Betulinic acid (mg/g)	3.25 ± 0.18*	2.77 ± 0.15

Data are presented as mean ± SD (*n* = 3). * denotes significant difference in ZSS and ZSS-Pi groups (*p* < 0.05).

**Table 2:** HPLC calibration curves for spinosin, jujuboside A, jujuboside B, and betulinic acid.

Compound	Linear Equation ^1^	Linear Range (μg/mL)	*R* ^2^
Spinosin	*Y* = 2,077,367 *X* + 346,638	27.40~2192	0.9998
Jujuboside A	*Y* = 2,029,085,549 *X* + 7380	12.60~1008	0.9998
Jujuboside B	*Y* = 248,640,993 *X* + 2455	12.50~1003	0.9996
Betulinic acid	*Y* = 6,967,454 *X* + 31,766	13.26~1058	0.9999

^1^ *Y* and *X* express the peak area and corresponding injection concentration, respectively.

## Data Availability

All relevant data generated or analyzed during this study are included in this published article and Supplementary Materials. Other raw data are available upon request.

## Supplementary Materials

The following supporting information can be downloaded at: https://www.mdpi.com/article/10.3390/metabo15030188/s1, Table S1: VOCs detected in ZSS and ZSS-Pi groups with HS-SPME-GC-MS.; Table S2: Detailed information of 21 differential metabolites between ZSS and ZSS-Pi groups.
