# Supplementary material for: Comprehensive Analysis on Physicochemical Properties and Characteristic Compounds of Insect-Infested Ziziphi Spinosae Semen
Source: Metabolites. 2025 Mar 11;15(3):188. doi: 10.3390/metabo15030188 (PMC11944026; doi:10.3390/metabo15030188)
Supplement: Supplementary file 1 [file metabolites-15-00188-s001.zip › Table S1.pdf]

Table S1. VOCs detected in ZSS and ZSS-Pi groups with HS-SPME-GC-MS.

No.	Volatile Organic Compounds	CAS	Formula	Molecular weight	Retention Time (min)	Relative average content (%, *n* = 3)	
						ZSS	ZSS-Pi
**Aldehydes**							
1	Phenylacetaldehyde	122-78-1	C_8H_8O	120	10.564	1.13±0.41	0.46±0.06
2	1-Nonanal	124-19-6	$C_9H_{18}O$	142	11.935	1.50±0.42	0.31±0.05*
3	Decyl aldehyde	112-31-2	$C_{10}H_{20}O$	156	14.608	0.27±0.07	0.05±0.01*
4	2,4-Dimethylbenzaldehyde	15764-16-6	$C_9H_{10}O$	134	14.931	0.88±0.23	0.42±0.05*
5	γ-Nonanolactone	104-61-0	$C_9H_{16}O_2$	156	19.633	3.64±0.63	0.85±0.19*
**Ketone**							
6	2-Decanone	693-54-9	$C_{10}H_{20}O$	156	14.243	3.38±2.31	0.37±0.26
**Alcohols**							
7	2,3-Butanediol	513-85-9	$C_4H_{10}O_2$	90	4.306	6.32±0.68	1.60±0.13*

8	Phenylethyl alcohol	60-12-8	$C_8H_{10}O$	122	12.174	1.55±0.33	0.27±0.05*
9	2-Phenoxyethanol	122-99-6	$C_8H_{10}O_2$	138	15.048	4.69±4.41	3.07±1.53
**Esters**							
10	Methyl octanoate	111-11-5	$C_9H_{18}O_2$	158	12.421	0.23±0.08	0.09±0.00
11	Methyl noanote	1731-84-6	$C_{10}H_{20}O_2$	172	15.142	2.06±0.44	1.81±0.08
12	Methyl decanoate	110-42-9	$C_{11}H_{22}O_2$	186	18.317	0.62±0.07	0.70±0.03
13	Methyl 9-oxo-nonanoate	1931-63-1	$C_{10}H_{18}O_3$	186	22.146	0.04±0.01	0.12±0.01*
14	Dimethyl suberate	1732-09-8	$C_{10}H_{18}O_4$	202	22.630	0.01±0.01	0.06±0.01*
15	Methyl 10-methylundecanoate	5129-56-6	$C_{13}H_{26}O_2$	214	25.429	2.29±0.66	0.49±0.09*
16	Dimethyl azelate	1732-10-1	$C_{11}H_{20}O_4$	216	26.294	0.02±0.01	0.18±0.02*
17	Methyl myristate	124-10-7	$C_{15}H_{30}O_2$	242	32.614	0.20±0.15	0.65±0.02*
18	Methyl pentadecanoate	7132-64-1	$C_{16}H_{32}O_2$	256	36.038	0.37±0.12	1.46±0.10*
19	Methyl palmitoleate	1120-25-8	$C_{17}H_{32}O_2$	268	38.663	0.16±0.05	1.88±0.06*

20	Methyl palmitate	112-39-0	$C_{17}H_{34}O_2$	270	39.579	21.79±5.00	40.46±1.24*
21	Ethyl palmitate	628-97-7	$C_{18}H_{36}O_2$	284	41.538	0.47±0.09	0.36±0.07*
22	Methyl oleate	112-62-9	$C_{19}H_{36}O_2$	296	45.182	0.20±0.13	5.21±0.21
23	Methyl stearate	112-61-8	$C_{19}H_{38}O_2$	298	45.683	0.24±0.12	1.17±0.05*
24	Methyl linoleate	112-63-0	$C_{19}H_{34}O_2$	294	46.161	0.09±0.06	0.51±0.03*
25	Ethyl elaidiate	6114-18-7	$C_{20}H_{38}O_2$	310	46.800	0.04±0.01	0.04±0.01
26	Methyl 10-trans,12-cis-octadecadienoate	21870-97-3	$C_{19}H_{34}O_2$	294	46.920	0.10±0.08	0.57±0.09*
27	Methyl 11-cis-eicosenoate	2390-09-2	$C_{21}H_{40}O_2$	324	50.585	0.02±0.01	0.32±0.06*
28	Methyl 8-methylnonadecanoate	65301-91-9	$C_{21}H_{42}O_2$	326	51.315	0.01±0.00	0.12±0.02*
**Hydrocarbons**							
29	Dodecane	112-40-3	$C_{12}H_{26}$	170	14.427	4.77±2.40	1.04±0.27
30	Tridecane	629-50-5	$C_{13}H_{28}$	184	17.501	7.17±2.57	1.36±0.42*
31	Tetradecane	629-59-4	$C_{14}H_{30}$	198	20.920	6.99±0.93	0.70±0.36*

32	Pentadecane	629-62-9	$C_{15}H_{32}$	212	24.521	6.39±1.28	0.87±0.55*
33	Hexadecane	544-76-3	$C_{16}H_{34}$	226	28.145	ND	1.19±0.61
34	Heptadecane	629-78-7	$C_{17}H_{36}$	240	31.704	3.09±0.16	0.97±0.48*
35	Octadecane	593-45-3	$C_{18}H_{38}$	254	35.153	0.91±0.05	0.35±0.16*
**Others**							
36	Azulene	275-51-4	$C_{10}H_8$	128	14.044	5.34±0.63	3.22±0.26*
37	1-Ethoxy-4,4-dimethyl-2-pentene	55702-60-8	$C_9H_{18}O$	142	14.842	0.56±0.30	0.08±0.01
38	2-Methyl-naphthalene	91-57-6	$C_{11}H_{10}$	142	17.347	0.72±0.01	0.44±0.05*
39	N,N-Dibutylformamide	761-65-9	$C_9H_{19}NO$	157	17.630	2.92±2.58	0.61±0.57
40	[(Hexadecyloxy)methyl]-oxirane	15965-99-8	$C_{19}H_{38}O_2$	298	24.403	ND	0.05±0.01
41	(Z)-13-Docosenamide	112-84-5	$C_{22}H_{43}NO$	337	52.065	0.47±0.41	0.28±0.12

* denotes significant difference of ZSS and ZSS-Pi group ($p < 0.05$).

The 41 VOCs were classified into six categories, including 19 esters, 7 hydrocarbons, 5 aldehydes, 3 alcohols, 1 ketone, and 6 other compounds (Figure 2B and Table 3). The content of 29 VOCs was significantly different in content between ZSS and ZSS-Pi group ($p < 0.05$).
