# Supplementary material for: Comprehensive Analysis on Physicochemical Properties and Characteristic Compounds of Insect-Infested Ziziphi Spinosae Semen
Source: Metabolites. 2025 Mar 11;15(3):188. doi: 10.3390/metabo15030188 (PMC11944026; doi:10.3390/metabo15030188)
Supplement: Supplementary file 1 [file metabolites-15-00188-s001.zip › Table S2.pdf]

Table S2. Detailed information of 21 differential metabolites between ZSS and ZSS-Pi groups.

No.	Retention time (min)	Metabolites	Formula	CAS	Scan mode	*m/z*	Mass error (ppm)	VIP
1	1.361	D-Proline	$C_5H_9NO_2$	344-25-2	+	116.0708	-3.45	3.12
2	1.546	Choline	$C_5H_{13}NO$	62-49-7	+	104.1072	-2.88	2.05
3	1.841	Hypoxanthine	$C_5H_4N_4O$	68-94-0	+	137.0459	-2.92	5.56
4	2.178	L-Pyroglutamic acid	$C_5H_7NO_3$	98-79-3	+	130.0501	-2.31	5.38
5	2.526	Adenosine	$C_{10}H_{13}N_5O_4$	58-61-7	+	268.1041	-1.86	3.21
6	6.675	D-(+)-Tryptophan	$C_{11}H_{12}N_2O_2$	153-94-6	+	205.0972	-2.44	2.56
7	15.135	Hexadecanamide	$C_{16}H_{33}NO$	629-54-9	+	256.2634	-2.34	9.21
8	15.253	Oleamide	$C_{18}H_{35}NO$	301-02-0	+	282.2790	-2.48	24.15
9	15.600	Stearamide	$C_{18}H_{37}NO$	124-26-5	+	284.2947	-2.11	9.81
10	1.210	L-Glutamic acid	$C_5H_9NO_4$	56-86-0	–	146.0458	+3.42	3.75

11	1.241	Gluconic acid	$C_6H_{12}O_7$	526-95-4	–	195.0508	+1.54	4.06
12	1.390	Uric acid	$C_5H_4N_4O_3$	69-93-2	–	167.0210	+2.99	6.40
13	1.392	Stachyose	$C_{24}H_{42}O_{21}$	470-55-3	–	665.2150	+1.50	6.48
14	1.410	Sucrose	$C_{12}H_{22}O_{11}$	57-50-1	–	341.1086	+0.59	12.46
15	1.493	L-Histidine	$C_6H_9N_3O_2$	71-00-1	–	154.0621	+2.60	2.21
16	1.629	D-Raffinose	$C_{18}H_{32}O_{16}$	25954-44-3	–	503.1615	+0.60	2.76
17	2.023	Xanthine	$C_5H_4N_4O_2$	69-89-6	–	151.0259	+1.99	2.94
18	4.852	Azelaic acid	$C_9H_{16}O_4$	123-99-9	–	187.0973	+1.60	2.90
19	12.129	Jujuboside A	$C_{58}H_{94}O_{26}$	55466-04-1	–	1205.5946	-0.75	2.74
20	14.763	cis-5,8,11,14,17-Eicosapentaenoic acid	$C_{20}H_{30}O_2$	10417-94-4	–	301.2169	+0.33	2.65
21	15.842	Deoxycholic acid	$C_{24}H_{40}O_4$	83-44-3	–	391.2853	+1.28	3.77
